# Monogenetic autoinflammatory syndromes and nephrology - therapy is usefull even in advanced kidney failure

**DOI:** 10.1186/1546-0096-13-S1-P5

**Published:** 2015-09-28

**Authors:** K Hohenstein-Scheibenecker, A Schmidt

**Affiliations:** 1 Medical University of Vienna, Nephrology, Vienna, Austria

## Objectives

The identification of genes involved in the modulation of inflammatory processes has allowed the delineation of a new group of diseases called “Monogenetic Autoinflammatory Syndromes - MAISs”.

At the moment, 25 syndromes and their gene-disorders are known. Some of them are well known (eg Familial Mediterranean Fever), most of them are rare diseases (eg FCAS-Familial Cold-Autoinflammatory Syndrome, Blau Syndrome, HIDS-Hyperimmuno-globulinemiaD with Periodic Fever Syndrome, MA-Mevalonate Aciduria).

These disorders of innate immunity characterized by episodes of fever and systemic inflammatory symptoms affect for instance the serosal surfaces and bear the risk of developing reactive systemic (AA) amyloidosis due to excessive production of serum amyloid-A (SAA). SAA is deposited in various organs, particularly the kidneys, with the consequent progressive development of kidney failure[^1^,^2^].

## Case 1 and 2

### Blau Syndrome

We report a now 26-year old patient, who was first admitted aged 5 months due to periodic fever. Additional patient's history included polyarthritis, uveitis and a worsening of renal function over the years due to histological signs of sarcoidosis in the kidney. Different immunosuppressive treatments with steroids, azathioprin, methotrexat and cyclosporin did not prevent renal failure requiring dialysis in 2005. In 2010 our patient underwent renal transplantation. Despite standard triple immunosuppression patient's symptoms, especially uveitis, occurred even after transplantation whenever steroids were reduced. The initial diagnosis “juvenile sarcoidosis” was corrected to “Blau Syndrome” due to mutation analysis carried out in April 2012 (mutation on NOD2 gene). Thanks to treatment with adalimumab since 2012, uveitis and polyarthritis are under control. Kidney function has remained stable with creatinin values within normal range up to this day.

### Familial Mediterranean Fever

A 54-year old dialysis patient with atrophic kidneys (reason unknown) underwent renal transplant in October 2012. A few days after discharge a fever of unknown origin occurred with an increase of c-reactive protein (CRP), creatinin, severe abdominal pain and diarrhoea without detection of pathogens. As Familial Mediterranean Fever (FMF) was suspected patient underwent a colchicin therapy. The creatinin, CRP and SAA value decreased at normal range within 6 weeks of treatment. To confirm the diagnosed FMF, a mutation analysis was carried out. We found a heterocygous mutation in the MEFV gene, which confirms the FMF diagnosis.

## Case 3

### Familial Cold-Autoinflammatory Syndrome

We report a women born in 1971 who was diagnosed with reduced kidney function in 2008. In addition the patient has been suffering from cold-dependent urticaria and joint pain without known reason since childhood. There was a rapid worsening of renal function over the next months. The kidney biopsy shows signs of amyloidosis (picture a-f). Mutation analysis in June 2013 confirms the diagnose “Familial Cold-Autoinflammatory Syndrome - FCAS”. Due to treatment with anakinra, the cold-dependenturticaria and joint pain are eliminated, SAA is normalized and serumkreatinin and proteinuria are decreased.

## Conclusion

Monogenetic Autoinflammatory Syndromes are rare causes of kidney failure due to excessive production of serum amyloid-A (SAA). The detection by means of mutation analysis allowed a disease-specific therapy with stabilization of serumcreatinin and SAA in addition to reduced disease-specific symptomes even in advanced kidney failure and after renal transplantation.

## Consent to publish

Written informed consent for publication of their clinical details was obtained from the patient/parent/guardian/relative of the patient.
